# Calcium signaling and postsynaptic density dynamics: the roles of calmodulin in synaptic protein regulation

**DOI:** 10.3389/fnmol.2026.1831452

**Published:** 2026-06-02

**Authors:** Keyaa Dilip Shah, Yonghong Zhang

**Affiliations:** School of Integrative Biological and Chemical Sciences (SIBCS), University of Texas Rio Grande Valley, Edinburg, TX, United States

**Keywords:** calcium, calmodulin, postsynaptic density, synaptic proteins, synaptic regulation

## Abstract

Calcium signaling in neuronal cells is the principal mechanism underlying learning and synaptic plasticity, whereas the postsynaptic density (PSD) is an important center where calcium-dependent processes are organized and regulated. This review explores the roles of calmodulin (CaM) in facilitating calcium-dependent modulation of the activities of synaptic proteins in the PSD. We focus on the molecular processes through which CaM interacts with calcium signals to regulate the function, localization, and interactions of central synaptic proteins, including calcium/calmodulin-dependent protein kinase II (CaMKII), membrane-associated guanylate kinases (MAGUKs), and N-methyl-D-aspartate (NMDA) receptors. We aim to summarize recent advances in structural, biochemical, and imaging studies of how CaM’s structural flexibility, calcium binding kinetics, and protein-protein interactions uncover complex regulatory loops that allow exquisite temporal and spatial control over synaptic efficacy. Additionally, research progress on how disruptions of CaM-mediated signaling pathways are linked to neurological diseases, which may be conducive to new potential therapeutic interventions, is discussed.

## Graphical Abstract

**Figure:**
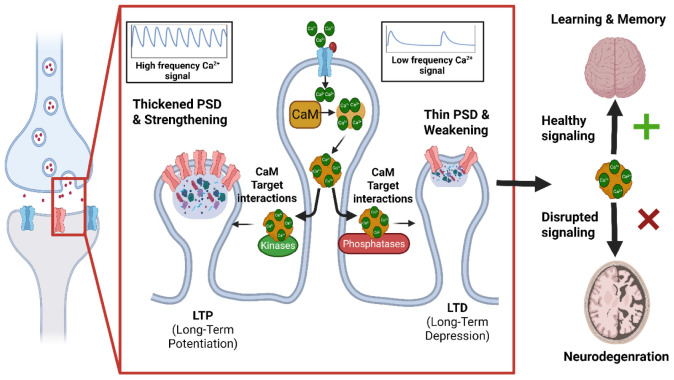
Illustration showing how calcium influx at excitatory synapses activates calmodulin (CaM), which decodes spatiotemporal Ca^2+^ signals to regulate postsynaptic density (PSD) organization, receptor dynamics, and downstream signaling pathways, thereby controlling synaptic plasticity and function.

## 1 Introduction

Chemical synaptic connections are specialized neuronal cell junctions for interneuron communication, comprising a presynaptic axon terminal, a synaptic cleft, a postsynaptic membrane, and a membrane-associated protein-rich region just underneath the excitatory postsynaptic membrane known as the postsynaptic density (PSD). The PSD region is highly organized and hosts more than 1500 different proteins, which are essential in forming complex signaling networks (Wilkinson and Coba, 2020). This intricate network of receptors, kinases, phosphatases, scaffolding proteins, and cytoskeletal components forms the fundamental scaffold on which neuronal proteins read and respond to incoming synaptic activity (Bayés et al., 2011).

The PSD is a dynamic structure that undergoes activity-dependent remodeling by changes in protein composition and organization, leading to variations in synaptic strength and stability (Sheng and Hoogenraad, 2007).

The synaptic adaptations are heavily dependent on intracellular calcium ions (Ca^2+^) signaling as the core messaging system transmitting transient synaptic activity to sustained molecular and architectural modifications. The entry of Ca^2+^ into the dendritic spines upon cell excitation occurs through surface receptors like N-methyl-D-aspartate receptors (NMDARs) and voltage-gated calcium channels (VGCCs), and intracellular stores. These sources generate spatially and temporally distinct calcium signals that activate downstream signaling pathways in the PSD. The differences in the outcomes from signaling cascades govern whether there will be strengthening of synapses, as in Long-term Potentiation (LTP), or reduced strengths, like in Long-term Depression (LTD), depending on the pattern and magnitude of signaling. These mechanisms are carried out by different proteins in the PSD in response to calcium signals and are central to cognitive functions of the brain, like learning and memory (Hayashi, 2022; Nicoll, 2017).

Among the various calcium-binding proteins in the PSD that relay the signal downstream, CaM is a remarkably resourceful and evolutionarily conserved messenger. It is a small (148 amino acids, 17 kDa) ubiquitous protein that consists of two approximately symmetrical globular domains joined by a short flexible linker, each containing two EF-hand motifs binding Ca^2+^. Its structural ability to undergo intense conformational changes upon calcium binding allows it to interact with over 300 known target proteins, thereby acting as a crucial transducer of calcium signals.

The calcium-binding properties of its N- and C-terminal lobes allow CaM to act as a calcium decoder by responding to diverse calcium signals, translating the frequency and scale of Ca^2+^ oscillations into specific outcomes (Cheung, 1980; Chin and Means, 2000).

In the PSD, CaM is not only a calcium sensor but also acts as a molecular switch that coordinates the activities of ion channels, scaffolding proteins, and regulators like kinases and phosphatases (Villalobo et al., 2018; Yang and Tsai, 2022). Ca^2+^-bound CaM acts as a regulator of processes, e.g., phosphorylation and dephosphorylation, which modulates the function of critical postsynaptic proteins like CaMKII, calcineurin (CaN), and scaffolding proteins such as PSD-95 and PSD-93 (Chin and Means, 2000; Wayman et al., 2008). Through these interactions, CaM influences many processes in the PSD, including receptor clustering, kinase activity, and dynamics of cytoskeletal structures (Chen et al., 2021). Understanding the regulatory roles of CaM is gaining massive significance, as disruptions in the CaM signaling pathway have been associated with neurological disorders like Parkinson’s disease, Alzheimer’s, and Autism spectrum disorders (Kaizuka and Takumi, 2018).

Recent advances in structural and experimental methodologies have provided new insights into CaM-mediated signaling. High-resolution imaging and microscopic techniques have revealed nanoscale organization of PSD proteins, while structural techniques, including Nuclear Magnetic Resonance (NMR), and cryo-electron microscopy (cryo-EM), are beginning to capture CaM–protein interactions in extreme detail (Villalobo et al., 2018). Furthermore, proteomic analysis has identified multiple scaffolding and other protein members of the PSD as targets of Ca^2+^-bound CaM, highlighting its regulatory role within the synaptic signaling networks (Vetter and Leclerc, 2003; Yang and Tsai, 2022).

In this review, we explore the multifaceted roles of CaM as a regulator of calcium signaling within the postsynaptic density. We incorporate the current findings on the structural organization of the PSD, the biochemical mechanisms of Ca^2+^ sensing by CaM, and its roles in key protein interactions. We also highlight emerging concepts like structural remodeling of the PSD and distinct calcium signals being translated to functional outcomes. Together, these insights illustrate how CaM can act as a key player in regulating the molecular machinery of plasticity and cognition.

## 2 Calcium signaling in neurons and its impact on synaptic function

In the nervous system, Ca^2+^ serves as the most vital secondary messenger that regulates many neuronal processes from the release of neurotransmitters to changes in the strength of the synapse, leading to learning, memory, and cognitive effects (Augustine et al., 1987). Changes in intracellular Ca^2+^ concentration in the dendritic spines of neurons play an important role in determining the fate of the processes occurring in synaptic transmission, i.e., LTP and LTD, which can either strengthen or weaken the synaptic activity (Berridge, 1998). The regulation of Ca^2+^ influx, its spatiotemporal distribution, and expulsion from the cell is therefore central to healthy neuronal communication.

### 2.1 Mechanisms of calcium entry into neurons

Upon neuronal excitation, Ca^2+^ ions enter the intracellular space of the postsynaptic neuron through various specialized sources. The NMDA-type glutamate receptors (NMDARs) act as both ligand-gated and voltage-dependent channels and represent a major entry source for Ca^2+^. Their activity is tightly regulated by Mg^2+^ binding, thus ensuring that the influx of Ca^2+^ ions starts only when glutamates are released from the presynaptic neuron, and the post-synaptic membranes are depolarized. Once activated, NMDARs allow the inflow of Ca^2+^ directly into dendritic spines, producing localized areas of elevated Ca^2+^ ion concentrations that trigger downstream signaling cascades (Paoletti et al., 2013). The Ca^2+^ signal produced in such a way is relatively slow and long-lasting, making it crucial for initiating long-term plasticity as required for memory formation and learning (MacDermott et al., 1986). In contrast, α-amino-3-hydroxy-5-methyl-4-isoxazolepropionic acid receptors (AMPARs) are primarily permeable to Na^+^ and K^+^ ions and mediate fast excitatory synaptic transmission; they also contribute to Ca^2+^ influx and modulate downstream signaling pathways (Liu and Zukin, 2007).

In addition to NMDARs, L, N, and P/Q type VGCCs contribute to Ca^2+^ entry during strong depolarization events or back-propagating action potentials. These signals last even longer and are often coupled with longer changes like gene-transcription modulations (Zhang et al., 2021). Moreover, intracellular storage of calcium, like the endoplasmic reticulum (ER), also supplies Ca^2+^ ions to the cytosol through their membrane receptors like inositol 1,4,5-trisphosphate receptors (IP3Rs) and ryanodine receptors (RyRs). Ca^2+^ is released from the ER in response to an initial rise in cytosolic calcium levels after synaptic activation by a process called calcium-induced calcium release (CICR), which results in further amplification of the Ca^2+^ signal (Berridge et al., 2003). Together, these sources create calcium hotspots in the dendritic spines, which modulate the amount, duration, and outcome of the calcium-linked signaling networks. Modern techniques like two-photon calcium imaging have revealed that a large hotspot of Ca^2+^ can be triggered by only a short synaptic stimulation that lasts only tens of milliseconds but can have overwhelming downstream effects (Sabatini et al., 2002).

### 2.2 Spatio-temporal regulation of calcium signals

The effect of calcium signals not only depends on the cytosolic concentration of Ca^2+^ ions but also on their duration (temporal) and location (spatial) in the cytosol. Experimental and computational studies on calcium kinetics have shown that brief, high-amplitude spikes of Ca^2+^ are produced in LTP events, whereas LTD is associated with lower-amplitude but sustained elevations (De Koninck and Schulman, 1998). Calcium decay experiments using calcium buffering proteins, like parvalbumin and calbindin, show differences in molecular target activation. Since CaM binds to Ca^2+^ with high affinity and rapidly changing binding kinetics, it acts as a versatile Ca^2+^ sensor and a strong decoder of calcium signals, translating calcium dynamics into specific biochemical outcomes. Imaging studies, such as FRET (fluorescence resonance energy transfer), have shown that CaM is indeed not uniformly distributed within the PSD but is localized to specialized regions, particularly concentrated near the cytoplasmic surface and fringe of the PSD, containing scaffolding proteins and receptors. This uneven distribution ensures that only specific signaling pathways are activated in response to calcium influx (Aoki et al., 2018), indicating that Ca^2+^ signals activate selective pathways rather than a widespread, non-specific response.

### 2.3 Calcium-dependent modulation of PSD

The PSD is a highly dynamic structure that continuously undergoes changes, including its size, shape, and molecular composition, in response to neuronal activity, in particular, calcium signals. Ca^2+^, as an essential intracellular signal that triggers neurotransmitter release, dictates the architecture and molecular composition of the PSD.

Ca^2+^ influx influences actin-binding proteins such as cofilin and gelsolin through CaM-dependent activation of kinases, e.g., myosin light chain kinase (MLCK) (Okamoto et al., 2004). These proteins play a key role in shaping the dendritic spine morphology, converting thin spines into mushroom-shaped ones associated with stronger synapses.

Along with changing surface area, calcium signaling also influences the receptor concentration on the membrane by regulating exocytosis and endocytosis of AMPA receptors as well as assembly and disassembly of scaffolding complexes in the PSD. These processes contribute to activity-dependent changes in PSD size and composition, which are associated with different synaptic strengths, providing a basis for transitioning between strong and weak synapses. (Grienberger and Konnerth, 2012; Shepherd and Huganir, 2007).

### 2.4 Calcium signals and synaptic plasticity

Synaptic plasticity is a process in which synaptic strengths are modulated to produce desired outputs like LTP and LTD. These outputs are the basis of learning, memory, and cognitive functions. Calcium signals are important biochemical triggers that determine the fate of plasticity. When the concentration of Ca^2+^ is high but transient, Ca^2+^/CaMKII is activated, which has been associated with the phosphorylation of AMPA receptors and an increase in their surface localization, to promote LTP (Lisman et al., 2012). However, some studies suggest that phosphorylation of specific AMPA receptor sites may not be strictly required for LTP, indicating that additional or compensatory mechanisms contribute to synaptic strengthening (Lee H. K. et al., 2010). On the other hand, moderate and sustained elevation in Ca^2+^ concentration activates phosphatases, like CaN and protein phosphatase 1 (PP1), which dephosphorylate the target proteins, thereby promoting LTD (Malenka and Bear, 2004).

**FIGURE 1:**
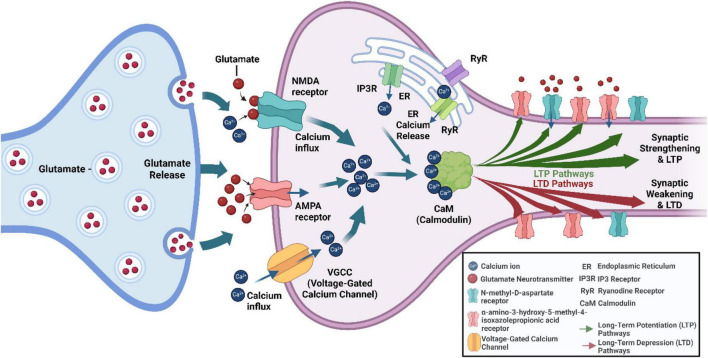
Overview of calcium signaling in the postsynaptic density (PSD). Graphical representation of calcium entry and signaling in a dendritic spine. Glutamate released from the presynaptic terminal activates postsynaptic N-methyl-D-aspartate (NMDA) and AMPA receptors, allowing calcium influx primarily through NMDA receptors and, under depolarized conditions, through voltage-gated calcium channels (VGCCs). Additional calcium is released from intracellular stores such as the endoplasmic reticulum (ER) through Ryanodine receptors (RyRs) and inositol-1,4,5-trisphosphate receptors (IP_3_Rs). These combined sources generate localized and temporally dynamic calcium hotspots. Local calcium elevations activate CaM, which interprets calcium signals to regulate downstream signaling pathways that can promote either LTP or LTD, thereby regulating synaptic strength.

These opposing pathways show how the time and concentration of the calcium signal can produce two completely opposite outcomes in the same neuron. The PSD provides a highly organized platform where these calcium-dependent signaling events are integrated, enabling efficient coordination of molecular responses to synaptic activity (Figure 1; Pavlidis et al., 2000).

## 3 Postsynaptic density: structure and key components

The PSD is one of the most complex and highly synchronized protein structures in the mammalian nervous system, acting as a highly dynamic, protein-rich molecular platform at excitatory synapses. Electron microscopy first revealed it as an electron-dense region residing beneath the postsynaptic membrane of excitatory synapses, appearing as a dark band (Palay, 1956). Beyond its visual density, the PSD is an extensive network for neurotransmitter receptors, scaffold proteins, cytoskeletal components, and signaling enzymes that together coordinate synaptic activity. Proteomic analyses have estimated that the PSD is comprised of more than 1,000–1,500 unique proteins (Song et al., 2024), rendering the PSD one of the largest protein structures in the nervous system. Importantly, the PSD is not a static structure but a highly dynamic and activity-dependent protein assembly that is continuously remodeled in response to synaptic activity. These structural and molecular changes are closely associated with differences in synaptic strength, suggesting that variations in PSD organization may underlie the distinction between weak and strong synapses (Sheng and Hoogenraad, 2007). This morphological and biophysical complexity is the biochemical basis enabling the PSD to act as a platform to integrate synaptic transmission, plasticity, and, consequently, cognition (Husi et al., 2000).

### 3.1 Molecular architecture and organization

**FIGURE 2:**
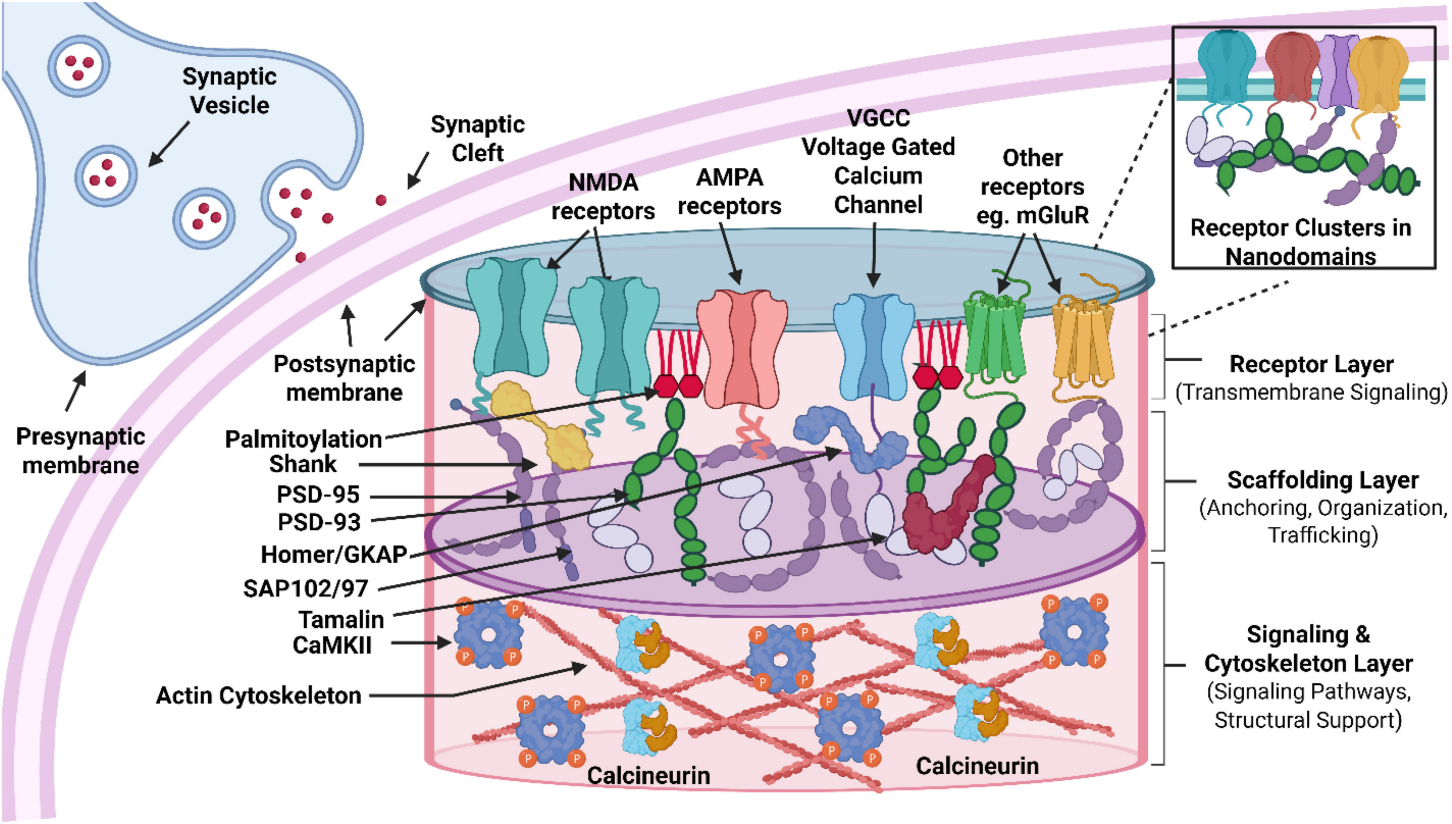
Structure and components of the postsynaptic density. Three-dimensional schematic illustrating the architecture of the postsynaptic density (PSD) beneath the postsynaptic membrane. Although depicted in layers for clarity, the PSD represents a highly interconnected and dynamic network rather than strictly segregated domains. The upper layer contains glutamate receptors, including N-methyl-D-aspartate (NMDA) and AMPA receptors, often organized into nanodomains to mediate synaptic transmission and calcium influx. The middle layer is enriched in scaffolding proteins such as PSD-95, PSD-93, SAP97, and SAP102, as well as additional scaffold proteins including Shank, Homer, GKAP, PICK1, Tamalin, and Norbin, which anchor receptors and organize signaling complexes. PSD-95 also interacts with the membrane through palmitoylation. The lower layer comprises signaling enzymes, including CaMKII and CaN, and small GTPases coupled to the actin cytoskeleton that supports structural plasticity.

Despite its complexity, the PSD has a highly ordered and regulated architecture (Figure 2). PSD proteins are organized in the form of isolated layers that roughly extend up to 30–50 nm into the cytoplasm (Harris and Weinberg, 2012; Palay, 1956). High-resolution microscopic studies have shown that each layer represents a distinct function (Bayés et al., 2017). The layers closer to the membrane are enriched with neurotransmitter receptors like AMPARs, NMDARs, and Kainite receptors, along with their scaffolding proteins. In contrast, the deeper layers contain secondary messengers, signaling molecules, and cytoskeletal elements that mediate downstream signaling and structural remodeling. This spatial organization enables efficient coupling between receptor activation and intracellular signaling pathways, allowing rapid and localized responses to synaptic activity.

In this framework, membrane-associated guanylate kinases (MAGUKs) comprising PSD-95, PSD-93, SAP-102, and SAP97 are the scaffolding proteins that recruit and stabilize glutamate receptors on the postsynaptic membrane and maintain their concentration on the membrane surface, and furthermore, link the receptors to the intracellular machinery. In addition to MAGUKs, several other scaffolding proteins like Shank, Homer, GKAP, PICK1, Tamalin, and Norbin contribute to PSD organization by forming interconnected networks that regulate receptor trafficking, signaling, and cytoskeletal dynamics (Romorini et al., 2004). These scaffolding proteins facilitate the rapid reconfiguration of the PSD in response to synaptic activity (Funke et al., 2005). The regulatory function is carried out by enzymes like kinases (CaMKII, PKA, PKC), phosphatases (PP1, CaN), and small GTPases such as Ras, Rap, and Rho family proteins (Sheng and Hoogenraad, 2007). Such biophysical organization and structural pattern of the most important “organ” in the neuron represents a base for moderating synaptic plasticity and signal transmission. These proteins work in harmony to continuously modulate and change the responses to synaptic activation.

### 3.2 Core components and their functions

#### 3.2.1 NMDA and AMPA receptor complexes

The PSD is built intracellularly around ionotropic glutamate receptors, mainly NMDA and AMPA receptors, located directly embedded in the postsynaptic membranes of excitatory synapses. NMDA receptors are heterotetrameric complexes composed of GluN1 and GluN2 (A-D) subunits that are physically linked with the PDZ domains of scaffolding proteins (Sheng and Kim, 2011). They are the central effectors for the inflow of Ca^2+^, and by interacting with different enzymes, they actively regulate the downstream signaling cascades (Berridge, 1998). In addition, NMDAR subunit composition influences receptor kinetics and calcium permeability, thereby contributing to differences in synaptic signaling and plasticity outcomes (Paoletti, 2011). AMPA receptors, in contrast, are very dynamic and mediate fast excitatory synaptic transmission and quickly move into and out of the PSD to the synaptic membrane surface. This process is regulated by AMPAR auxiliary subunits like TARPs and the PDZ-binding motifs that interact with PSD-95 and other scaffold proteins (Huganir Richard and Nicoll Roger, 2013). Under certain conditions, such as the absence of the GluA2 subunit, AMPARs can also become permeable to Ca^2+^, thereby contributing directly to calcium signaling and synaptic plasticity (Liu and Zukin, 2007). The coordinated regulation of NMDAR-mediated calcium influx and AMPAR trafficking is central to activity-dependent changes in synaptic strength. Several other receptors on the surface of PSD include metabotropic glutamate receptors (mGluRs) and adhesion molecules (neuroligins, cadherins). This further increases the tight regulation of functions inside the PSD.

#### 3.2.2 Scaffolding proteins

Postsynaptic density -95 (*DLG4*) is the most extensively studied member of the MAGUK scaffolding family and plays a central role in organizing synaptic components. It interacts directly with the GluN2 subunits of the NMDA receptors and TARPs of AMPA receptors and acts to anchor them in the postsynaptic membrane (Chen et al., 2011). PSD-93 (*DLG2*) is a homolog of PSD-95 and also plays a key role in stabilizing receptor complexes on the postsynaptic membrane (Citri and Malenka, 2008). Both proteins have similar backbone structures, containing three PDZ domains, an SH3 domain, and a guanylate kinase-like (GK) domain. However, knockout studies reveal that they show variations in regions governing non-redundant functions (Fitzjohn et al., 2006). There are some other MAGUKs that contribute to developmental and activity-dependent regulation of receptor localization. SAP102 is significantly abundant during early postnatal development, essential for trafficking of NMDARs and AMPARs, synapse formation and maturation, while SAP97 interacts strongly with AMPA receptor subunits during synaptic activity-dependent modulations (Metzbower et al., 2024).

In addition to MAGUK proteins, several other scaffolding proteins are essential for PSD organization and synaptic plasticity, including Shank, Homer, GKAP (SAPAP), PICK1, Tamalin, and Norbin. These proteins form interconnected networks that link receptors to intracellular signaling pathways and the actin cytoskeleton. For example, GKAP links PSD-95 to Shank proteins, which in turn interact with Homer proteins to connect glutamate receptors to intracellular signaling complexes and cytoskeletal elements (Romorini et al., 2004).

These MAGUKs and associated scaffold proteins act as hubs for synaptic remodulation and plasticity as well as downstream signaling. For example, during LTP, PSD-95 undergoes phosphorylation and palmitoylation under the effect of Ca^2+^/CaM, which regulates its association with the membrane and receptor anchorage (Ho et al., 2011). On the other hand, synaptic silencing causes them to declutter and thereby reduces the synaptic presence of receptors. These constant modifications show how these MAGUK proteins coordinate PSD architecture to adjust to incoming signals.

Together, these scaffolding proteins provide a framework that ensures the proper arrangement of receptors, allowing efficient synaptic signaling.

#### 3.2.3 Signaling proteins and enzymes

The PSD is a hub for hundreds of secondary messengers and enzymes participating in a network of signaling cascades. One of the most abundant signaling proteins, comprising about 2% of the total protein content in the PSD, is CaMKII. CaMKII functions both as a kinase and as a structural protein. CaMKII binds and interacts with multiple proteins, like CaM, NMDA receptors, and other small signaling molecules. Being a kinase, it acts by phosphorylating members of signaling cascades and receptors upon activation (Lisman et al., 2002). Another common mediator of calcium signals in the PSD is CaN, which dephosphorylates key proteins and acts in synaptic weakening (Mulkey et al., 1994).

Some small GTPases are very crucial in the PSD to function in association with MAGUKs to regulate the surface expression of receptors. Synaptic GTPase-activating protein (SynGAP) interacts tightly with the PDZ domains of PSD-95 and controls signaling in the MAPK pathway (Sheng et al., 2004). SynGAP is phosphorylated by CaMKII in a Ca^2+^/CaM-dependent manner by CaMKII, resulting in its rapid dispersion from the PSD of dendritic spines during LTP. This SynGAP dispersion is a crucial step that relieves its inhibitory effect on Ras, leading to increased synaptic Ras activity, subsequently, AMPA receptor insertion into the synaptic membranes, and synaptic strengthening.

This coordinated regulation of kinases, phosphatases, and GTPases enables efficient translation of calcium signals into long-term changes in synaptic strength. The colocalization of these signaling molecules to the receptors and downstream effectors in the PSD provides an efficient way to translate calcium signals to synaptic plasticity, underlying the major cellular mechanisms of learning, memory, and cognition.

#### 3.2.4 Cytoskeletal structural components

The PSD proteins are closely associated with the actin cytoskeleton of dendritic spines to bring structural changes in response to requirements. Synaptic activation and Ca^2+^ signals are relayed to structural modulation of the spines via actin-binding proteins like cortactin, cofilin, and α-actinin, which regulate actin polymerization and depolymerization. These cytoskeletal dynamics help to provide a way to enlarge the spines for LTP and shrink them for LTD (Hotulainen and Hoogenraad, 2010). Scaffold proteins further coordinate these structural changes. Mutational studies have discovered that GKAP (guanylate kinase-associated protein) acts as a bridge between Shank and the Homer family of proteins, which directly modulate the actin cytoskeleton (Tu et al., 1999). This coupling between scaffold proteins and the actin cytoskeleton provides a mechanistic basis for how calcium-dependent signaling can drive long-term structural modifications of dendritic spines. Defects and dysfunctions in these protein interactions are connected to disorders like the autism spectrum and Alzheimer’s disease (Durand et al., 2007).

### 3.3 Dynamic behavior of the PSD

The PSD was initially thought to be a relatively rigid structure, but imaging and biochemical studies have shown that it is very dynamic, as a continuous exchange of proteins exists between its membranes and cytoplasmic pools, as revealed by live cell imaging (Zheng et al., 2010). During synaptic plasticity, many scaffolding proteins were also observed to be highly mobile in an activity-dependent manner, causing rapid reorganization of the PSD composition. This continuous mobility is regulated by post-translational modifications on proteins like palmitoylation/depalmitoylation, ubiquitination, and phosphorylation/dephosphorylation, etc., that control protein localization, stability, and interactions (El-Husseini Ael et al., 2002). These modifications ensure the rapid remodeling of protein networks in the PSD, making it invariably adaptable to neuronal activity. As a result, the PSD functions as a highly adaptable structure capable of undergoing continuous reorganization in response to synaptic signaling.

### 3.4 Functional significance of the PSD

Postsynaptic density integrates and amplifies the signals in the neurons by providing a functional zone for receptors, scaffolding proteins, post-translational modification enzymes, neurotransmitters, and other components. This helps in transforming localized chemical signals into long-lasting synaptic changes. The organization of PSD functions as an operational network for multiple protein interactions to occur (Dosemeci et al., 2016). Ultimately, the PSD is not just a static collection of proteins but a molecular machine, continuously adapting to changing conditions in the synaptic membranes and translating this adjustment into long-term adaptations in the neuron via effector proteins such as CaM. Importantly, the PSD serves as a molecular interface where transient calcium signals are translated into sustained biochemical and structural changes, linking synaptic activity to long-term neuronal adaptation. Thus, rather than being a static collection of proteins, the PSD operates as a dynamic molecular machine that continuously adapts to synaptic activity, with effector molecules such as CaM playing a central role in coordinating these processes.

## 4 Calmodulin as a master regulator of PSD proteins

The PSD hosts a variety of calcium-binding proteins, one of which is CaM. CaM, being a versatile protein, acts as a mediator of Ca^2+^ signals. CaM functions as a key transducer of Ca^2+^ signals by binding calcium and undergoing conformational changes that enable its interactions with numerous molecular targets. It not only buffers the signals but also helps in decoding the signal levels by responding to their amplitude, duration, and frequency, thereby linking calcium dynamic signals with various molecular targets (Chin and Means, 2000). CaM regulates many ion channels to control cellular calcium entry, it also induces dynamic changes in scaffolding proteins, thereby influencing synaptic strength and plasticity.

### 4.1 Structural and biochemical features of calmodulin

As a small acidic protein, CaM is evolutionarily significant and is highly conserved across eukaryotes (Cheung, 1980). It consists of two globular lobes (N- and C-terminal lobes), each containing two EF-hands for calcium-binding. These lobes are connected by a flexible central linker, which allows CaM to be essentially flexible and undergo conformational changes, exposing hydrophobic surfaces that facilitate interactions with target proteins (Chin and Means, 2000).

The two lobes have varying intensities of binding affinity for Ca^2+^: the C-terminal lobe has a higher affinity and slow kinetics for binding calcium, while the N-terminal lobe binds with lower affinity and faster kinetics (Xia and Storm, 2005). This unequal binding affinity allows CaM to react selectively to a wide range of calcium signals, from rapid fluctuations to slow elongated releases.

NMR structural studies have demonstrated that once CaM binds Ca^2+^, it can engage with various protein targets due to its large conformational change to expose its hydrophobic pockets. This mechanism allows it to interact with a variety of targets (Hoeflich and Ikura, 2002). Unbound CaM (apo) can also interact with specific protein targets, which provide it with an additional regulatory function (Villalobo et al., 2018).

### 4.2 Calmodulin as a calcium sensor in neurons

The calcium concentration in the PSD constantly fluctuates in the form of oscillations, waves, and transient spikes. Upon activation, the calcium levels near the synaptic membrane can reach up to micromolar concentrations, leading to the formation of microdomains that selectively activate calcium sensors in the PSD (Kennedy and Weissbourd, 2024). There are many calcium sensors in the neuron, like parvalbumin or calbindin, which act in buffering the calcium released. Unlike them, CaM doesn’t reduce the signal; it transduces that signal to downstream processes. CaM can also be pre-associated with certain targets even before synaptic activation and Ca^2+^ release. These “primed complexes” can immediately respond upon calcium entry (DeMaria et al., 2001).

The activation of CaM depends on the amplitude and frequency of Ca^2+^ signals. For example, high-frequency calcium spikes preferentially activate CaMKII, promoting LTP, while lower, sustained elevations of Ca^2+^ may instead activate CaN, leading to LTD (Lisman et al., 2002; Malenka and Bear, 2004). However, these relationships are not absolute, and additional signaling components and molecular context influence the final synaptic response. CaM can dictate different results emerging from synaptic activity and calcium signals owing to this dual mode of action, which makes it a very powerful molecular switch.

Studies by imaging techniques reveal that CaM is not equally distributed throughout the neuron but is usually targeted to activated synapses. FRET analysis has shown transient immobilization of CaM near the PSD during periods of synaptic activity (Lee K. H. et al., 2010). This is likely due to CaM binding to its target proteins and receptors upon calcium influx in the PSD (Aoki et al., 2018). CaM organizes the flow of information from the synapse to other areas of the neuron by binding to different targets in a Ca^2+^ dependent manner. Such spatial confinement ensures selective activation of signaling pathways without widespread cross-activation within the neuron.

### 4.3 Regulation of calmodulin within the PSD

Although CaM acts classically as an effector of calcium signals, its function is also subject to multiple regulatory influences. Modifications such as phosphorylation, acetylation, and oxidation tightly regulate its calcium-binding affinity, interactions with other proteins, and localization within the PSD (Erickson et al., 2008). Even the local concentration of active CaM protein is regulated by CaM-binding proteins like neurogranin and CaM-dependent kinase kinase (CaMKK) that act as “reservoirs” to sequester CaM under resting conditions and release it only during synaptic activation (Zhabotinsky et al., 2006). Recent imaging studies show that CaM is not distributed evenly in the PSD but enriched in domains near calcium entry sites, ensuring spatial specificity of signaling (Kang et al., 2020).

Calmodulin’s regulatory flexibility also allows feedback loops for signaling. For example, CaMKII, once activated by Ca^2+^-bound CaM, phosphorylates CaM-binding proteins, thereby modulating downstream signaling pathways. These regulatory mechanisms promote fine-tuning of CaM-dependent signaling pathways in response to calcium signals with high specificity (Tsui and Malenka, 2006).

### 4.4 Calmodulin at the crossroads of synaptic function

**FIGURE 3:**
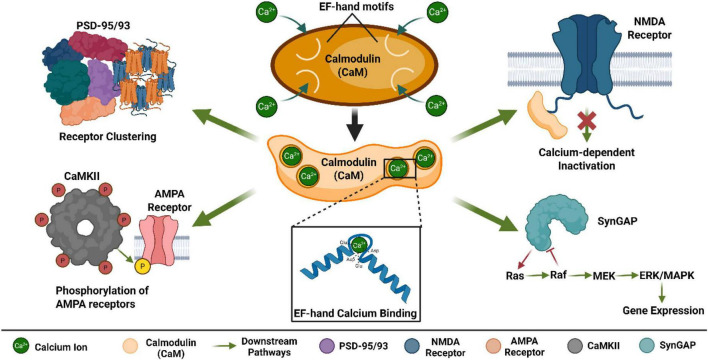
Calmodulin functions as a molecular switch in postsynaptic signaling. Calmodulin (CaM) undergoes a calcium-dependent conformational change upon binding Ca^2+^ ions at its four EF-hand motifs. Activated Ca^2+^/CaM interacts with multiple synaptic targets to deduce the calcium signals, including PSD-95 to regulate receptor clustering, N-methyl-D-aspartate (NMDA) receptors to mediate calcium-dependent inactivation, CaMKII to drive AMPA receptor phosphorylation, and SynGAP to modulate Ras–MAPK signaling.

Calmodulin acts as an important convergence point for multiple signaling pathways contributing to synaptic modulation in the PSD. This is probably due to the vast variety of interactions it undergoes upon activation. As shown in Figure 3, CaM targets a wide range of proteins involved in diverse synaptic processes through direct or indirect interactions, like receptor regulation (NMDA, AMPA, SK channels) to enzymatic control (CaMKII, CaN, adenylyl cyclases) and structural plasticity (PSD-95, actin-binding proteins) (Wayman et al., 2008).

Calmodulin plays a central role in shaping the biochemical outcomes of calcium signals, although these outcomes are also influenced by additional signaling pathways and molecular context. Whether a synapse is undergoing LTP or LTD depends in part on what CaM-dependent pathway is being activated. Its interactions with key PSD scaffolding proteins such as PSD-93 and PSD-95 will be discussed in the next section highlighting how molecular recognition and structural changes translate into changes in synaptic stability, receptor distribution, and ultimately, memory formation.

## 5 Calmodulin’s interactions with key PSD proteins

Calmodulin modulates the synaptic function through direct as well as indirect interactions with a large number of target proteins in the PSD. Among these targets, some are central to synaptic organization, like the MAGUK family scaffolds, e.g., PSD-95 (*DLG4*) and PSD-93 (*DLG2*). Through interaction with these scaffolding proteins leads to indirect subsequent adaptation in the overall structural organization of the PSD. Other indirect interactions occur via synaptic enzymes that catalyze phosphorylation and other modifications. These interactions provide a mechanistic link between transient calcium signals and longer-lasting structural and functional changes within synapses. This section discusses key interactions of CaM involved in PSD regulation.

### 5.1 Calmodulin and MAGUK scaffolds (PSD-95/PSD-93)

Postsynaptic density -95 and PSD-93 are prototypical MAGUK scaffold proteins that are pivotal in aligning NMDA and AMPA receptor complexes through the PDZ domains with the postsynaptic membrane and connecting downstream effector molecules via the SH3 and GK domains (Sheng and Kim, 2011). Earlier pull-down assays and yeast two-hybrid screening showed that CaM binds PSD-95 in a calcium-dependent manner, but the exact binding site was not known until recently (Baron et al., 2006).

Recent NMR studies, mutagenesis, and palmitoylation assays by Zhang et al. (2014), Chowdhury et al. (2018), Chowdhury and Hell (2019) show that Ca^2+^/CaM binds the N-terminal and HOOK regions of PSD-95, interfering with its palmitoylation. Ca^2+^/CaM binding competes with the palmitoylation modification at the sites of Cys3 and Cys5, thereby inducing PSD-95 to disperse from the postsynaptic membrane, which causes a reduction in AMPA receptor clustering (Chowdhury and Hell, 2019; Chowdhury et al., 2018; Zhang et al., 2014). These experiments establish a molecular connection between Ca^2+^ spikes and long-lasting alterations in scaffolding functions that drive LTD-like mechanisms. However, the extent to which CaM-mediated PSD-95 depalmitoylation alone is sufficient to drive LTD remains an area of ongoing investigation, suggesting that additional pathways may contribute to these processes.

NMR binding studies done by Fukunaga et al. (2005) found that CaM also binds a specific region in the PDZ2 domain on PSD-95 when activated by calcium signals. This interaction interferes with ligand binding to PSD-95, like TARP, and SynGAP, reducing their binding affinity (Fukunaga et al., 2005). Some other peptides, like Pyk2, binding to the SH3-GK domains of PSD-95, are also modulated by CaM. Pull-down assays done by McGee et al. (2001) and immunoprecipitation studies by Bartos et al. (2010) helped show that under elevated calcium levels, CaM associates with the SH3-GK domain of the protein (Chetkovich et al., 2002). However, a detailed structure for this association has yet to be studied.

The interaction between PSD-93 and CaM is not well-studied. Interestingly, new biochemical and computational work suggests that CaM might also associate with non-canonical sites within the SH3 domain and the PDZ1-2 region of PSD-93 (Paarmann et al., 2008). These interactions are weaker, but they appear to influence the intra-domain dynamics and protein clustering at the PSD.

These interactions may modulate protein clustering and scaffold flexibility within the PSD, although their functional significance requires further investigation (Opazo et al., 2012). Collectively, CaM interactions with MAGUK scaffolds can regulate receptor clustering, scaffold stability, and downstream signaling pathways, thereby linking calcium transients to structural changes in synaptic organization.

### 5.2 Calmodulin and receptor regulation

NMDA receptors (NMDARs) are both the source of activity-dependent Ca^2+^ influx in the dendritic spine and a direct target for CaM. Ca^2+^ influx through NMDARs generates local transients that recruit CaM, which then binds to intracellular portions of NMDAR subunits (GluN1 and GluN2B), while also interacting with PSD-95, thereby creating a “CaM–NMDAR–PSD-95 signaling triad.” This triad causes calcium-dependent inactivation (CDI), which modulates downstream signaling (Rafiki et al., 1997; Rameau et al., 2000). CDI is a negative-feedback mechanism that prevents excess Ca^2+^ entry in the neuron and excitotoxic signaling. CaM binding induces conformational changes in the receptor that regulate channel activity and downstream signaling (Zhang et al., 1998). Through this mechanism, CaM shapes both the magnitude and duration of calcium signaling within dendritic spines.

Calmodulin-mediated modulation of NMDARs also influences interactions between receptors and scaffolding proteins such as PSD-95, thereby affecting the coupling of receptors to intracellular signaling pathways (Ehlers et al., 1996b).

Recent high-resolution structural and functional studies have significantly advanced our understanding of how CaM regulates NMDA receptor (NMDAR) activity in a calcium-dependent manner. Using a combination of NMR spectroscopy, isothermal titration calorimetry (ITC), and electrophysiological recordings, it was demonstrated that Ca^2+^-bound CaM directly interacts with cytosolic C-terminal regions of both GluN1 and GluN2A subunits, providing a molecular basis for Ca^2+^-dependent channel desensitization (CDD). Functional disruption of these interactions through targeted mutations greatly reduced CDD, suggesting that CaM binding to GluN1 and GluN2A converges on a shared regulatory mechanism. Together, these findings support a model in which multiple Ca^2+^/CaM molecules bind to the intracellular domains of the NMDAR, forming a negative feedback loop that limits excessive Ca^2+^ influx while maintaining synaptic plasticity (Bej et al., 2026). However, the relative contribution of CaM-mediated receptor modulation compared to other regulatory mechanisms remains an active area of investigation.

### 5.3 Calmodulin-driven phosphorylation/dephosphorylation cascades

Apart from binding to the membrane proteins and altering the PSD structural stability, CaM also functions by activating or deactivating certain kinases and phosphatases, which regulate signaling cascades and long-term molecular changes in the PSD.

Calmodulin-dependent protein kinase II is a major kinase in the PSD and acts both enzymatically by phosphorylating substrates like GluA1, stargazin, etc., and structurally binds to NMDAR subunits and scaffolds to support synaptic activity (Lisman et al., 2012). Structural studies by X-ray crystallography and kinetic assays reveal that Ca^2+^/CaM binding relieves autoinhibition of CaMKII and causes its activation; subsequent autophosphorylation at Thr286 provides partial autonomy (Chang et al., 2017). This leads to a prolonged biochemical signal even after calcium levels drop in the neuron. Research by single-molecule FRET and atomic force microscopy indicates that the holoenzyme undergoes conformational changes (Chang et al., 2017). CaMKII upon activation by Ca^2+^/CaM, phosphorylates AMPAR subunits (increasing conductance), stargazin/TARP proteins (promoting PSD-95 interaction and synaptic retention), and actin-regulatory proteins to modulate synaptic strength for LTP generation (Derkach et al., 1999). However, genetic and physiological studies suggest that phosphorylation of specific AMPAR sites (e.g., GluA1 Ser831) may not be strictly required for LTP, indicating the presence of compensatory mechanisms and additional regulatory pathways (Rellos et al., 2010).

CaN and other phosphatases are also activated by Ca^2+^/CaM during LTD induction. Synapse stimulation by low-frequency pulses leads to Ca^2+^/CaM activation of CaN, as evidenced by patch-clamp studies, which dephosphorylates inhibitor-1, leading to PP1 activation and AMPAR dephosphorylation and sequestration (Mulkey et al., 1994). Recent reviews are emphasizing questions about CaN activation and CaN kinetic factors that will be important for targeting phosphatases therapeutically (Williams and Johnson, 2021). These opposing kinase and phosphatase pathways illustrate how CaM translates calcium dynamics into bidirectional changes in synaptic strength.

SynGAP, a Ras GTPase-activating protein, is not directly activated by CaM but by CaM-dependent kinases like CaMKII (Kim et al., 1998). Studies from fluorescence imaging and mass spectrometry have indicated that after LTP induction, SynGAP is phosphorylated by CaMKII, reducing its GAP activity toward Ras and increasing Ras/ERK signaling pathway that promotes AMPAR insertion, supporting further LTP stability and spine morphology (Walkup et al., 2015).

### 5.4 Ion channels and auxiliary regulators

Calmodulin also directly regulates other ion channels and sensors closely tied to the PSD function. For example, SK (small conductance Ca^2+^-activated K^+^) channel gating is dependent on CaM acting as a sensor to activate the channel (Lee and MacKinnon, 2018). VGCCs are also mediated by CaM. Patch-clamp demonstrates that CaM mediates calcium-dependent inactivation in L-type and P/Q-type channels, tuning presynaptic release and postsynaptic Ca^2+^ entry (DeMaria et al., 2001). In these feedback mechanisms, CaM functions as both a decoder and a controller of calcium influx sources. In addition, Other auxiliary proteins like neurogranin, IQGAP1, affect the availability of CaM in active form, thereby fine-tuning its binding to its target proteins and overall plasticity in the neuron (Putkey et al., 2024). These regulatory mechanisms further refine CaM signaling by controlling both its localization and functional interactions within the PSD.

Proteomics and interactome studies indicate that CaM interacts with a large number of proteins in neurons and other tissues (Villalobo et al., 2018). Mapping of CaM-binding proteins in the PSD using Mass Spectrometry is beginning to reveal how CaM’s signaling network is continuously adapted during plasticity, stress, and disease conditions. These datasets lead us to identify and study unrecognized CaM targets for therapeutic interventions.

Altogether, these studies display how CaM converts the multidimensional levels of Ca^2+^ signals (amplitude, duration, frequency, and spatial localization) into coordinated and long-lasting modifications of synaptic structure and function.

## 6 Roles of calmodulin in plasticity underlying learning and memory

The capacity of synapses to alter their strength in response to neuronal activity is known as synaptic plasticity, which is the molecular foundation of learning and memory. At the core of this mechanism primarily lies calcium signaling, with CaM acting as a key mediator. Acting as both a sensor and an effector, CaM translates small changes in the intracellular Ca^2+^ concentration into precise biochemical events that reshape the PSD, reorganize receptor populations, and regulate signaling networks.

This section integrates molecular mechanisms, structural and imaging evidence, and behavioral data to provide a comprehensive account of how CaM contributes to the induction, expression, and maintenance of LTP and LTD.

### 6.1 Calmodulin as a decoder of Ca^2+^ dynamics and plasticity

Calcium signals vary in amplitude, duration, and frequency, and CaM functions as a decoder of these variations by selectively activating downstream effectors. High local Ca^2+^ concentrations preferentially activate lower-affinity, fast-responding targets such as CaMKII, whereas lower and sustained Ca^2+^ levels favor activation of high-affinity targets such as CaN (Pepke et al., 2010; Pharris et al., 2019). Computational and experimental studies have shown that this frequency decoding arises from the interplay between the kinetics of CaM binding and release, CaMKII subunit cooperativity, and competing interactions between signaling pathways (Pharris et al., 2019).

**FIGURE 4:**
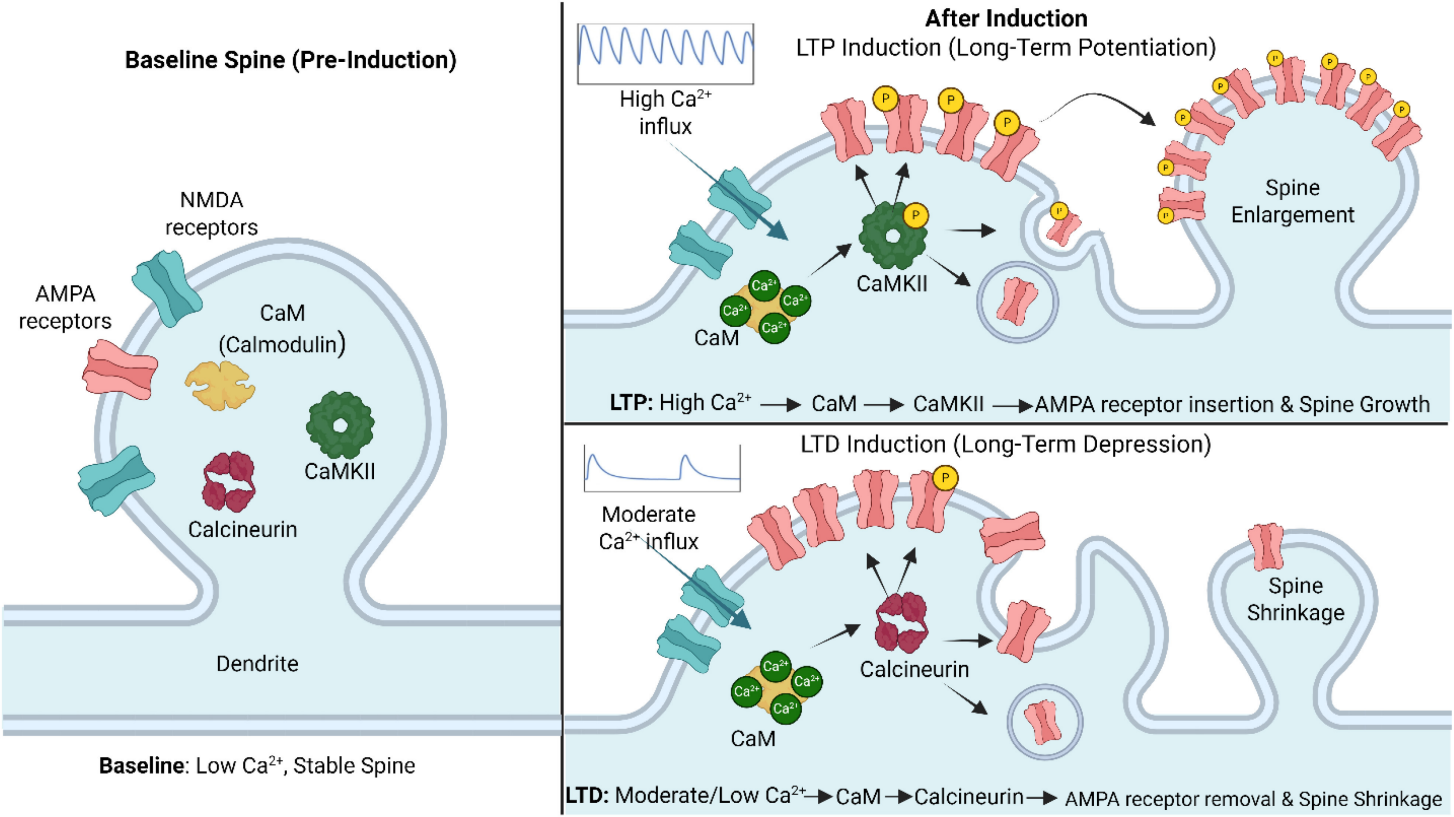
Calmodulin-dependent signaling in synaptic plasticity. Comparison of signaling pathways leading to long-term potentiation (LTP) and long-term depression (LTD) at excitatory synapses. High-amplitude, transient calcium influx through N-methyl-D-aspartate (NMDA) receptors activates Ca^2+^/CaM and CaMKII, promoting AMPA receptor phosphorylation and insertion and synaptic strengthening leading to LTP. In contrast, lower or sustained calcium elevations activate CaM-dependent phosphatases such as CaN, resulting in AMPA receptor internalization and synaptic weakening, leading to LTD.

The induction of LTP occurs via brief but high-intensity calcium spikes triggered by rapid local Ca^2+^ influx into the spine through NMDA receptors (Figure 4; MacDermott et al., 1986). These calcium spikes rapidly saturate CaM, leading to the activation of Ca^2+^/CaMKII. Structural studies using cryo-EM and single-molecule FRET have revealed that CaM binding induces a conformational rearrangement in CaMKII’s regulatory segment, relieving autoinhibition and facilitating autophosphorylation at residue Thr286 (Chao et al., 2011; Rosenberg et al., 2005). This phosphorylation converts CaMKII into a switch and retains its activity even after Ca^2+^ levels subside (Miller and Kennedy, 1986). Activated CaMKII then phosphorylates AMPA receptor subunits, particularly GluA1 at Ser831 and auxiliary proteins like TARP, thereby increasing receptor conductance and promoting their insertion in the membrane (Lee et al., 2000). However, studies using knock-in models have shown that phosphorylation of specific sites such as GluA1 Ser831 may not be strictly required for LTP, suggesting that compensatory mechanisms or parallel pathways contribute to synaptic strengthening (Rellos et al., 2010). This produces a persistent strengthening of the synaptic activity. Experimental disruptions of the CaM-dependent signaling pathway, like CaMKII Thr286 mutants and CaM antagonists, impair LTP and behavioral memory, suggesting the supporting role that CaM plays in cognitive function via LTP induction (Silva et al., 1992).

On the other hand, LTD arises when Ca^2+^ signals are weaker and more prolonged, resulting in preferential activation of Ca^2+^/CaM-dependent phosphatase CaN (Figure 4; Mulkey et al., 1994). CaM binding to CaN activates its enzymatic domain, leading to dephosphorylation of AMPA receptor subunits and downstream signaling molecules. This facilitates AMPA receptor internalization and spine surface reduction (Kim et al., 2007). Similar to LTP, LTD mechanisms are influenced by multiple signaling pathways, and CaM-dependent phosphatase activity represents one component of a broader regulatory network.

Thus, by selectively interacting with either CaMKII or CaN, depending on the Ca^2+^ spike frequency, CaM contributes to bidirectional regulation of plasticity on synapses. Computational models (Pepke et al., 2010) and pulse frequency manipulations confirm this frequency-dependent choice.

The structural state of the PSD is dynamically regulated by calcium signaling, with CaM playing a critical role in transitions between “thick/strong” and “thin/weak” synapses. During synaptic activity, elevated Ca^2+^ levels promote binding of Ca^2+^/CaM to key scaffolding proteins such as PSD-95. This interaction has been shown to interfere with PSD-95 palmitoylation at its N-terminal cysteine residues, leading to its destabilization and dissociation from the postsynaptic membrane. As PSD-95 serves as a major anchor for glutamate receptors, particularly AMPA receptors via TARPs, its removal results in reduced receptor clustering and weakening of synaptic signaling. Consequently, this process is associated with a thinning of the PSD structure and reduced synaptic efficacy, consistent with LTD-like states. In contrast, stabilization and accumulation of PSD-95 on the postsynaptic membrane and its associated scaffold complexes support receptor retention and enlargement of the PSD, contributing to thicker and stronger synapses. Thus, Ca^2+^/CaM-dependent modulation of scaffolding protein palmitoylation and receptor anchoring provides a mechanistic link between calcium signaling and activity-dependent structural remodeling of the PSD (Chowdhury and Hell, 2019; Chowdhury et al., 2018; Zhang et al., 2014).

### 6.2 Modulation of receptor trafficking

One of CaM’s most interesting roles is its influence on AMPA receptor trafficking, a process that determines the number of receptors at the postsynaptic membrane. As indicated in previous sections, AMPAR trafficking is coordinated by an interplay between stargazin (TARP-γ2), PSD-95, and auxiliary proteins (Tomita et al., 2005). Studies using live-cell imaging and labeling have demonstrated that increases in synaptic Ca^2+^ concentration trigger CaM-dependent phosphorylation cycles that maintain AMPARs at the synapse during LTP (Makino and Malinow, 2009).

As discussed in the previous section, CaM regulates NMDA receptor activity directly by binding to the cytoplasmic tails of GluN1 and GluN2B subunits. CaM mediates the feedback inhibition of NMDAR currents, preventing excitotoxic calcium overload (Ehlers et al., 1996a). However, during repeated stimulation, phosphorylation by CaMKII can displace CaM, increasing channel open time and facilitating synaptic potentiation.

Furthermore, CaM also modulates metabotropic glutamate receptor (mGluR)-dependent signaling pathways by coupling to adenylyl cyclases and phosphodiesterases, and it fine-tunes cyclic nucleotide signaling that connects with synaptic plasticity pathways (Wayman et al., 2008). Through these multiple regulatory mechanisms, CaM integrates receptor activity with intracellular signaling networks.

### 6.3 Structural remodeling: cytoskeleton

Apart from receptor modulation, CaM also serves as a molecular bridge between calcium signaling and the actin cytoskeleton, which regulates spine morphology and stability. Within dendritic spines, the majority of CaM’s targets are actin-associated proteins such as caldesmon, MLCK, and α-actinin (Sobue et al., 1982). CaM’s activation of MLCK drives myosin II-mediated contractility, contributing to the retraction and reshaping of spine ends during synaptic plasticity (Rubio et al., 2011).

Additionally, CaM also indirectly influences actin polymerization through its interaction with CaMKII and cofilin, promoting cytoskeletal polymerization during LTP and depolymerization during LTD (Okamoto et al., 2004). Super-resolution microscopy and two-photon imaging studies have revealed that calcium-induced spine enlargement coincides with a burst of CaM activity near the synapse (Harvey and Svoboda, 2007). These processes provide a structural basis for activity-dependent changes in synaptic strength.

### 6.4 Integration with PSD scaffolds and downstream signaling

The regulation of synaptic plasticity by Ca^2+^/CaM is closely linked to the PSD scaffold protein organization. As discussed in the previous section, CaM regulates the post-translational modification, localization, and conformations of MAGUK proteins such as PSD-95 and PSD-93. These scaffolds organize the molecular environment in which CaM’s downstream targets operate. For instance, the Ca^2+^/CaM-dependent release of PSD-95 from the postsynaptic membrane can temporarily destabilize receptor complexes, allowing for AMPAR insertion or removal at the appostsynaptic membrane. This structural rearrangement coincides with CaM-driven phosphorylation events to coordinate both the functional and architectural elements of plasticity (Zhang et al., 2014).

Calmodulin also regulates indirectly with the Ras-ERK and PI3K-Akt signaling pathways through effectors such as SynGAP, neurogranin, and CaMKK, as highlighted in the sections above. These cascades influence gene transcription in the nucleus via CREB phosphorylation, connecting synaptic calcium dynamics to long-term transcriptional changes affecting memory formation (Zhang et al., 2014). Thus, CaM provides a functional bridge between localized calcium signals at synapses and broader cellular responses, including gene expression changes associated with long-term neuronal adaptation.

## 7 Dysfunction of calmodulin-mediated signaling in neurological disorders

The discussion about CaM in this study is incomplete without exploring the detrimental effects when this highly specific machinery breaks down. Indeed, disruptions of Ca^2+^/CaM-mediated signaling have emerged repeatedly in models and human studies of neurodegenerative, psychiatric, and developmental disorders. These dysfunctions may appear from mislocalized CaM and its targets, altered binding affinities, mutations, irregular post-translational modifications, or imbalances between kinase and phosphatase activity. While CaM is not the sole driver of these pathologies, disruptions in Ca^2+^/CaM-dependent signaling pathways contribute significantly to synaptic dysfunction observed in disease states. The following sections highlight representative examples illustrating how alterations in CaM signaling are associated with neurological disorders.

### 7.1 Alzheimer’s disease and dementias

In Alzheimer’s disease (AD), one of the earliest and most common findings is synaptic dysfunction that comes before neuronal death. Part of this dysfunction appears to be implicated by altered CaM signaling. For example, in animal models (APP/PS1 mice) and in vascular dementia, researchers have observed altered levels of CaV1.2 (L-type VGCC subunit), CaM, phosphorylated CaMKII (p-CaMKII), CREB, and brain-derived neurotrophic factor (BDNF) in the hippocampus and cortex (Min et al., 2013). In some cases, CaM levels are increased, but downstream activation of p-CaMKII, p-CREB, and BDNF expression is reduced, indicating a mismatch between upstream signaling and downstream effect. In cultured neurons that are treated with amyloid-β, intracellular Ca^2+^ levels rise, and the signaling cascade becomes disturbed (Min et al., 2013). Some studies have exhibited significantly elevated CaM levels as compared to controls, detected via western blotting, when compared to cells from healthy individuals (Gunes et al., 2022).

Tau proteins, another hallmark of AD, have also been connected to CaM-dependent kinases and phosphatases. For instance, *in vivo* brain tissue analysis, biochemical assays of kinase/phosphatase activity, and postmortem studies of human hippocampal tissue indicate that CaMKII and CDK5 contribute to phosphorylation of tau at sites involved in tangle formation; while CaN (a CaM-dependent phosphatase), which normally reduces excessive tau phosphorylation, in AD, its regulation appears to be compromised (Ghosh and Giese, 2015).

There are several other instances that link AD to CaM targets, like PSD-95 and GluN2B subunits of NMDA receptors, which are reduced in AD models and cognitive tasks like spatial memory declines (Chowdhury and Hell, 2019). Interestingly, inhibitors of CaN, such as FK506, in transgenic mice restore PSD-95 levels, GluN2B, spine integrity, and LTP, suggesting that over-activation of the phosphatase pathway of CaM signaling might contribute to the pathology (Zeng et al., 2024).

### 7.2 Parkinson’s disease and neurodegeneration

Parkinson’s disease (PD) is associated with dopaminergic neuron loss. It also displays discrepancies in synaptic plasticity. In animal models of PD, dopamine loss leads to reduced levels of GluN1 subunits and PSD-95 in the PSD of striatal neurons, and increased autophosphorylation of CaMKII. These changes include increased recruitment of activated CaMKII to NMDA receptor complexes. Electrophysiological recordings in dopamine-denervated circuits, particularly within the striatum, demonstrate severe impairments in synaptic plasticity, with a loss of both LTP and LTD at corticostriatal synapses. Some pharmacological interventions, like inhibitors of CaMKII such as KN-93, have shown partial reversal of both synaptic and motor deficits, indicating that overactive or mislocalized CaMKII contributes to dysfunction (Picconi et al., 2004).

Additional studies in PD models show that oxidative stress can damage components of the CaM-CaMKII axis, possibly altering CaM’s ability to bind and regulate its targets. For example, modeling and *in vitro* studies suggest that oxidation of CaMKII or its misfolding may reduce CaM binding affinity, disrupting downstream signaling and contributing to synaptic destabilization (Pullara et al., 2024). These findings highlight the sensitivity of CaM-dependent signaling pathways to cellular stress conditions.

### 7.3 Developmental and psychiatric disorders

Apart from degenerative disease, function-impaired mutations of CaM or CaM-targeted proteins are linked to developmental and psychiatric conditions. The human genome carries three genes (*CALM1-3*) encoding CaM, and even a single amino acid substitution in any one allele disrupts CaM binding with voltage-gated ion channels or downstream effectors. These mutations have been identified in human epilepsies or intellectual disability syndromes, which might be linked to changes in calcium sensitivity or localization of CaM, leading to irregular excitability and impaired plasticity (Robison, 2014).

Calmodulin-dependent protein kinase II dysfunction has also been attributed to schizophrenia, epilepsy, and autism. Studies from immunohistochemistry and gene expression analysis have revealed that in some schizophrenia models, expression patterns of CaMKII isoforms are altered, and their subcellular localization is shifted, and downstream signaling, such as CREB activation, is reduced. These changes delineate a connection between CaM pathway disruptions and cognitive defects (Robison, 2014).

Mutations in other CaM-binding proteins are also relevant. For example, variants in Kv7 potassium channels reduce their CaM binding capacity, leading to altered membrane trafficking and increased neuronal excitability associated with epilepsy. Similar phenomena are observed with Nav1.2 sodium channel mutations in early-onset seizure disorders (O’Day, 2022).

### 7.4 Therapeutic implications

The aforementioned examples suggest that both hyper-activation and hypo-activation of CaM’s downstream interactors, like CaMKII and CaN, might lead to synaptic dysfunction. In contrast, blocking irregular components using phosphatase inhibitors like FK506 or CaMKII inhibitors can partially restore synaptic markers and plasticity.

Therapeutic strategies might also include small molecules or peptides that selectively enhance or stabilize CaM binding to specific targets such as PSD-95, GluN1/GluN2 subunits, or that rebalance kinase/phosphatase activity. Elevated CaM also might act as a biomarker for AD in peripheral cells, which may help early diagnosis and monitoring (Min et al., 2013).

**FIGURE 5:**
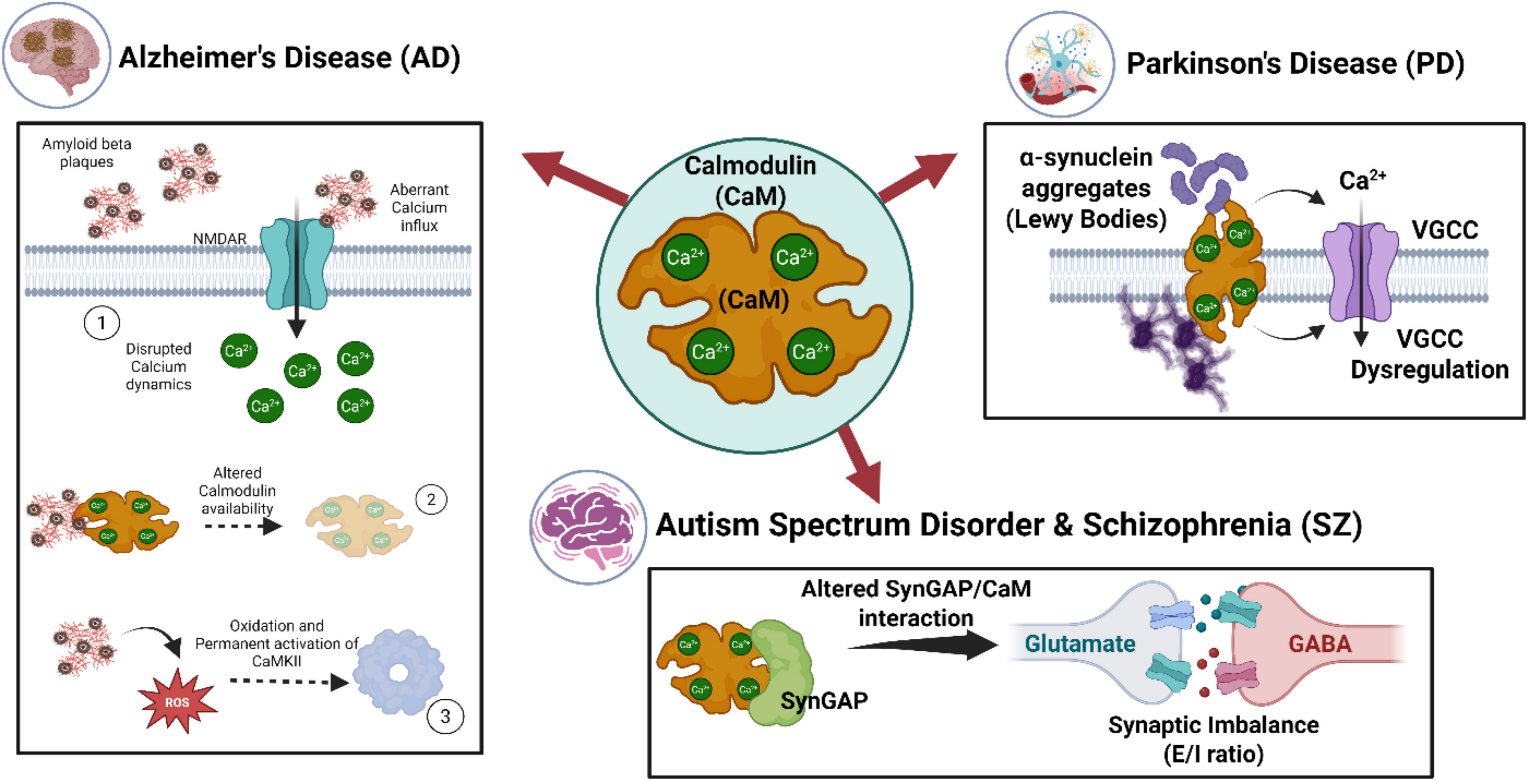
Calmodulin dysfunction in neurological disorders. Schematic view of how alterations in calmodulin (CaM)-dependent signaling contribute to neurological diseases. In Alzheimer’s disease (AD), amyloid-β oligomers interfere with CaM–CaMKII signaling directly and indirectly, impairing synaptic plasticity. In Parkinson’s disease (PD), α-synuclein aggregation disrupts calcium homeostasis and VGCC function. In autism spectrum disorders and schizophrenia, altered interactions between CaM and SynGAP lead to imbalances in Ras–MAPK signaling and synaptic regulation. Potential therapeutic strategies are aimed at stabilizing CaM interactions.

A deeper understanding of the molecular mechanisms underlying CaM dysfunction, as illustrated in Figure 5, may enable targeted modulation of CaM signaling pathways, offering potential avenues for therapeutic intervention.

## 8 Emerging research

Over the past decade, the understanding of the postsynaptic density has evolved from a relatively static, multiprotein-rich structure into a dynamic, continuous, synaptic activity-dependent structural remodeling organelle with calcium playing a key role in regulation. Advances in imaging technologies have enabled visualization, quantification, and manipulation of PSD components in living neurons with increasing spatial and temporal resolution. Traditional electron microscopy first revealed the PSD as a dense, fuzzy structure beneath excitatory synapses. Today, super-resolution fluorescence microscopy (STORM, PALM, and SIM) and cryo-electron tomography (cryo-ET) allow us to watch individual PSD components move and reorganize within nanometer ranges. CaM translocation, for instance, has been visualized as a rapid wave following NMDA receptor activation, coinciding with changes in spine surface area and receptor mobility (Sawada et al., 2018). These findings support a model in which multiple spatially restricted CaM–protein complexes interpret local calcium microdomains rather than a single uniform CaM pool mediating global responses. This emerging perspective refines our understanding of how synaptic plasticity may be encoded at the nanoscale level.

Proteomics has always been central to PSD biology, but recent crosslinking mass spectrometry (XL-MS) and proximity labeling (BioID, APEX) have begun to show interactions of CaM signaling. Using calcium-dependent affinity purification, investigators have identified over 400 CaM-binding proteins in synaptosomal fractions (Paget-Blanc et al., 2022). This includes canonical targets—CaMKII, neuromodulin, myosin V, but also unexpected partners such as metabolic enzymes and RNA-binding proteins that may localize temporarily to the PSD.

A parallel revolution is occurring in the use of synthetic biology to reconstitute PSD assemblies. Minimal PSD-like condensates formed from recombinant PSD-95, SynGAP, and NMDA receptor cytoplasmic tails are being used extensively (Pandey et al., 2025). Adding CaM to these systems reorganizes the droplets in a calcium-dependent manner, revealing that CaM might not only interpret calcium signals but also might physically remodel protein assemblies.

Transcriptomic datasets and machine learning analyses are being used to predict how developmental or disease-associated mutations in CaM-binding motifs might affect the entire PSD network.

### 8.1 Therapeutic and translational directions

Insights from molecular, structural, and computational studies of CaM signaling are beginning to inform potential therapeutic strategies. One path is the design of small molecules or peptides that specifically modulate CaM–target interactions rather than globally blocking CaM (Alaimo et al., 2013).

Another promising direction involves gene therapy and protein engineering. Since CaM is encoded by three identical genes (*CALM1-3*), CRISPR-based approaches can correct pathogenic mutations in specific isoforms without affecting others. Moreover, synthetic CaM variants with altered calcium sensitivity and binding affinities could serve as molecular tools to change neuronal sensitivity with high precision (Nyegaard et al., 2012; Prakash et al., 2022).

Finally, the possibility that CaM levels or modifications in peripheral cells reflect central nervous system dysfunction raises the prospect of biomarkers for early detection of synaptopathies. For example, altered CaM expressions have been reported in peripheral cells derived from patients with Alzheimer’s disease, although further validation is required (Corbacho et al., 2017). Continued investigation into CaM signaling mechanisms may enable the development of targeted therapeutic strategies aimed at restoring synaptic functions.

## 9 Conclusion

Postsynaptic density represents one of the most complex and specialized macromolecular protein machineries within neurons. With its highly organized structure and dynamic structural remodeling, it translates electrical signals into long-term changes in synaptic strength for neuronal communication. At the core of this mechanism lies calcium, a universal signaling messenger whose oscillations encode information regulating neuronal activity, and CaM that mediates these calcium signals into precise biochemical outputs through its ability to bind, modulate, and reorganize a vast array of target proteins. Rather than functioning as a single dominant regulator, CaM operates within a network of signaling pathways to modulate synaptic activity through interactions with a diverse set of target proteins. Throughout this review, we have highlighted how calcium and CaM-dependent signaling systems contribute to multiple layers of synaptic regulation, spanning from the interactions within the PSD to mechanisms underlying memory consolidation. The dual mode of CaM function enables neurons to discriminate between different patterns of calcium activity, driving selective downstream pathways to initiate either LTP or LTD. The PSD scaffolding proteins, particularly the PSD-95 MAGUK family, emerge as key proteins that contribute to CaM integrating calcium signaling with structural organization. By directly binding to these proteins, CaM indirectly influences receptor clustering, synaptic strength, and plasticity. This versatility elucidates how a single molecule can contribute to both synaptic potentiation and depression, and how it coordinates processes such as AMPA receptor trafficking, actin remodeling, and synaptic signaling.

Importantly, emerging evidence suggests that CaM not only decodes calcium signals but also participates in the dynamic reorganization of protein networks within the PSD, providing a mechanistic link between transient signaling events and long-term structural changes. From a clinical perspective, disruptions in CaM-mediated signaling have been implicated in a range of neurological and psychiatric disorders, including autism spectrum disorders, schizophrenia, epilepsy, and neurodegeneration. Mutations in CaM-binding motifs or alterations in calcium homeostasis might trigger cascading effects that destabilize synaptic architecture and impair plasticity. While therapeutic strategies targeting CaM signaling are still in early stages, advances in molecular and computational approaches offer promising avenues for future investigation.

Moving forward, integrating experimental, structural, and computational insights will be essential to fully understand how CaM-mediated signaling regulates synaptic function and contributes to brain physiology and disease.
